# A network perspective on cognition in individuals with Parkinson's disease

**DOI:** 10.1002/dad2.70091

**Published:** 2025-02-24

**Authors:** Daniel Scharfenberg, Elke Kalbe, Monika Balzer‐Geldsetzer, Daniela Berg, Rüdiger Hilker‐Roggendorf, Jan Kassubek, Inga Liepelt‐Scarfone, Brit Mollenhauer, Kathrin Reetz, Oliver Riedel, Sandra Roeske, Jörg B. Schulz, Alexander Storch, Claudia Trenkwalder, Karsten Witt, Hans‐Ulrich Wittchen, Richard Dodel, Anja Ophey

**Affiliations:** ^1^ Medical Psychology, Neuropsychology and Gender Studies Center for Neuropsychological Diagnostics and Intervention (CeNDI) University Hospital Cologne and Faculty of Medicine University of Cologne Cologne Germany; ^2^ Ethikkommission Ludwig‐Maximilians‐Universität München Munich Germany; ^3^ Department of Neurology University Medical Center Schleswig‐Holstein, Christian Albrechts‐University (CAU) Campus Kiel Kiel Germany; ^4^ Department of Neurology Klinikum Vest Recklinghausen Germany; ^5^ Faculty of Medicine Ruhr‐University Bochum Germany; ^6^ Department of Neurology University Hospital Ulm Ulm Germany; ^7^ Department of Neurodegenerative Diseases Hertie Institute for Clinical Brain Research and German Center for Neurodegenerative Diseases (DZNE) University Tübingen and German Center for Neurodegenerative Diseases Tübingen Germany; ^8^ Department of Psychology IB‐Hochschule Stuttgart Germany; ^9^ Department of Neurology Paracelsus‐Elena Klinik Kassel Germany; ^10^ Department of Neurosurgery University Medical Center Göttingen Germany; ^11^ Department of Neurology RWTH Aachen University Hospital Aachen Germany; ^12^ JARA Institute Molecular Neuroscience and Neuroimaging (INM‐11) Research Center Jülich Jülich Germany; ^13^ Department Clinical Epidemiology Leibniz Institute for Prevention Research and Epidemiology‐BIPS Bremen Germany; ^14^ German Center for Neurodegenerative Diseases (DZNE) Bonn Germany; ^15^ Department of Neurology University of Rostock and German Center for Neurodegenerative Diseases (DZNE) Rostock/Greifswald Rostock Germany; ^16^ Department of Neurology School of Medicine and Health Sciences and Research Center Neurosensory Science University of Oldenburg Oldenburg Germany; ^17^ Department of Neurology Evangelic Hospital Oldenburg Oldenburg Germany; ^18^ Center of Neurosensory Sciences University of Oldenburg Oldenburg Germany; ^19^ Clinical Psychology & Psychotherapy Ludwig‐Maximilians‐University Munich Munich Germany; ^20^ Department of Geriatric Medicine University Duisburg‐Essen Essen Germany

**Keywords:** cognitive decline, cognitive domains, dimensionality analysis, mutualism hypothesis, network analysis, network neuropsychology, Parkinson's disease

## Abstract

**INTRODUCTION:**

In neuropsychological diagnostics, the assignment of cognitive tests to domains is usually not empirically based. Hence, we aimed to assess the dimensionality structure of cognition in individuals with Parkinson's disease (PD) and conceptually replicate the findings in cognitively healthy individuals (CHIs).

**METHODS:**

We performed Exploratory Graph Analysis (EGA) for dimensionality analysis of cognitive test scores in *N *= 698 individuals with PD from the DEMPARK/LANDSCAPE study. Redundancy was reduced based on Unique Variable Analysis (UVA) before re‐performing EGA. CHI data (*N *= 60,398) served as a conceptual replication base.

**RESULTS:**

EGA identified five dimensions. After removing redundancy identified by UVA, EGA identified a unidimensional structure of cognitive test scores. The findings were conceptually replicated in CHIs.

**DISCUSSION:**

The findings imply the need to re‐evaluate the composition of cognitive test batteries to reduce redundancy and improve the validity of cognitive diagnostics. Cognition may be better described as a network of interrelated cognitive functions rather than a factorial structure of latent cognitive domains.

**Highlights:**

- Cognitive test scores of the same paradigm were strongly associated with each other.
- This finding indicates redundancy in the cognitive test battery.
- After removing redundancy, scores were best represented by unidimensional structures.
- The findings in Parkinson's disease were conceptually replicated in healthy controls.
- The results suggest that cognition should be viewed as a complex “network” of interrelated functions.

## 1 BACKGROUND

Although the diagnosis of Parkinson's disease (PD) is based primarily on motor symptoms, there is a large number of accompanying non‐motor symptoms, often including cognitive impairment.^1^, ^2^ Mild cognitive impairment (MCI), prevalent in 40% of individuals with PD,^3^ is characterized by cognitive performance below the age‐appropriate norm, but with little or no impact on activities of daily living. More severe cognitive impairment significantly impairing an individual's independence in activities of daily living is referred to as PD dementia (PD‐D).^4^

Given the high risk of cognitive impairment for individuals with PD and its implications for disease prognosis and therapeutic decisions, its valid and reliable diagnosis is essential. For diagnosing PD‐MCI, guidelines by the Movement Disorder Society (MDS) recommend a detailed neuropsychological examination across five cognitive domains (attention and working memory, executive functions, memory, language, and visuospatial functions) with at least two different tests each.^5^ PD‐MCI is diagnosed if at least two tests indicate impairment, that is, performance of at least one standard deviation (SD) below the appropriate norms. In addition, the MDS guidelines propose differentiating single‐domain (one cognitive domain impaired) and multiple‐domain PD‐MCI (two or more cognitive domains impaired)^5^ as well as amnestic (if memory is affected) and non‐amnestic PD‐MCI. With combining of these classifications, we can differentiate four PD‐MCI subtypes based on cognitive domains,^5^ which were found to be associated with different prognoses regarding further cognitive decline.^6^

Although cognitive domains play a crucial role in the MDS guidelines for diagnosing PD‐MCI and subtypes, the concept of cognitive domains is not well defined, as both definitions of cognitive domains and the assignment of cognitive functions or tests to cognitive domains differ. The diagnostic criteria for neurocognitive disorder in the fifth edition of the Diagnostic and Statistical Manual of Mental Disorders (DSM)^7^ define six domains (attention, executive functions, learning and memory, language, visual‐perceptive function, and social cognition); however, the MDS guideline suggests testing five domains, neglecting social cognition. In addition, the two guidelines differ in assignments of cognitive functions to domains; for example, regarding working memory (DSM: executive function; MDS guideline: attention) and verbal fluency (DSM: language; MDS guideline: executive function).^5^, ^7^ It is important to note that neither of the two definitions provides empirical justifications for their choice of categorization, providing no binding guidelines that map specific tests to cognitive functions and/or domains. As a result, assignments of tests to cognitive domains might not correctly reflect the underlying latent functions and challenge comparability between studies, thereby potentially limiting the reliability and validity of cognitive diagnoses and subtype classifications.

Given the heterogeneity of conceptually based assignments of cognitive tests to cognitive domains, data‐driven approaches to identify the cognitive domain structure in individuals with PD, like factor analysis (FA) methods, are important to ensure valid cognitive diagnostics. However, previous research has shown limitations of this approach when applied to healthy or mixed samples, because group‐specific characteristics might be neglected.^8^ It is notable that existing studies even in homogenous samples of individuals with PD could mostly not confirm previously assumed structures of cognitive domains across a range of different methods and assumed structures; for example, using Exploratory FA^9^ or Confirmatory FA (CFA) to test bi‐factor models of cognition.^10^ An alternative for FA methods is *exploratory graph analysis* (EGA).^11^ This advanced form of network analysis allows identification of a dimensionality structure within network models. EGA shows comparable or better performance than widely used FA methods^12^ and allows for a network perspective on cognition with the possibility of applying dimensionality analysis within the network. Two studies applied EGA to cognitive test scores of subgroups on the spectrum from groups of individuals with healthy cognition over subjective cognitive decline (SCD) and MCI to Alzheimer's dementia (AD), yielding inconsistent results in small sample sizes.^13^, ^14^ Existing studies that apply EGA to cognitive test scores do not fulfill simulation‐based sample size recommendations for EGA.^12^ Furthermore, EGA for cognitive test scores has not yet been applied in individuals with PD.

**RESEARCH IN CONTEXT**

1. **Systematic review**: The authors reviewed the empirical literature using common online databases (e.g., PubMed) to identify existing dimensionality analyses of cognitive functioning in Parkinson's disease (PD) as well as guidelines for diagnosing cognitive impairment in PD. The assignments of cognitive tests to cognitive domains for research and clinical applications were widely theoretically driven rather than empirically based, threatening the validity of cognitive diagnoses in PD.
2. **Interpretation**: The identification of a unidimensional structure of cognitive test scores following the removal of redundancy suggests a view of cognition as a complex system of interconnected cognitive functions instead of a factorial structure of latent cognitive domains.
3. **Future directions**: More studies that empirically analyze the structure of cognitive functioning in individuals with PD and other conditions are needed, to ensure the validity of cognitive diagnoses in PD and beyond. Ideally, such studies would apply network analysis methods to adequately reflect the complexity of cognitive functioning.

Psychometric network analysis of cognitive test scores is a relatively new field of research termed *network neuropsychology*,^15^ enabling us to analyze the complex interplay between cognitive functions operationalized as cognitive test scores. Test scores are represented as nodes in a network model, with edges between the nodes representing conditional associations between them.^16^ Network analysis is the underlying principle of many functional magnetic resonance imaging (fMRI) analyses,^17^ and it has helped to shift our perspective toward viewing the brain as a highly complex and interconnected system (rather than modular). It seems highly plausible that if the underlying anatomic features are represented as a network in the human brain, behavioral outcomes of assessed cognitive functions would be as well. Consequently, network analysis has been emerging recently as a method to analyze interconnections between cognitive functions as well.^15^ Furthermore, this method opens the possibility of discarding the idea of an underlying latent factor structure of cognitive domains, that is, a *general factor (g‐factor)* of intelligence,^18^ to model cognition in favor of a mutualism hypothesis waiving latent factors.^19^

In this study, we aim to empirically analyze the network and dimensionality structure of cognitive test scores in a sample of individuals with PD from the DEMPARK/LANDSCAPE cohort study^20^ with EGA. This approach enables us to compare the results to a previously assumed theoretical structure, both qualitatively and quantitively. Finally, we will critically evaluate the cognitive test battery used and validate our findings in a large sample of cognitively healthy individuals (CHIs) that was compiled in a factor meta‐analysis conducted by Agelink van Rentergem et al.^21^

## 2 METHODS

### 2.1 Participants

We analyzed the baseline data of individuals with PD from the *Dementia and Parkinson's disease*/*Langzeitbeobachtung dementieller Symptome und cognitiver Parameter sowie Anwendbarkeit neuer prognostischer Marker bei der Parkinson‐Erkrankung* (DEMPARK/LANDSCAPE) study,^20^ a completed, observational, prospective multi‐center cohort study that aimed to characterize the natural course of cognitive decline in individuals with PD. Participants were recruited consecutively from nine movement disorder centers across Germany. Inclusion criteria were age between 45 and 80 years and a diagnosis of idiopathic PD based on the UK Parkinson's Disease Society Brain Bank criteria. The study included individuals with cognitive functions within the demographically adjusted norms (PD‐NC), with PD‐MCI, as well as with PD‐D, following diagnostic criteria for cognitive impairment^22^, ^23^ available at the time of study set‐up. Of the original sample (*N *= 711), we excluded those without cognitive classification at baseline (e.g., due to missing data) or whose data for the cognitive variables of interest were completely missing. The final sample for data analysis consisted of *N *= 698 individuals with PD.

### 2.2 Cognitive and clinical assessment

An overview of the cognitive test battery and the corresponding theoretically assigned cognitive domains in the DEMPARK/LANDSCAPE study^24^ are reported in Table 1. Domain assignment was based on experts’ opinions. Raw cognitive test scores were demographically adjusted and standardized using published normative data (e.g., to percentiles, *z*‐scores, or *T*‐scores). Subsequently, these standardized scores were uniformly transformed into *z*‐scores. For descriptive statistics and distributions of cognitive test scores, see Figure S1. In addition, the data include sociodemographic variables (age, gender, years of education), clinical variables (disease duration; levodopa equivalent daily dose [LEDD]; Unified Parkinson's Disease Rating Scale Part III [UPDRS‐III]^25^; Hoehn & Yahr stages^26^; Geriatric Depression Scale [GDS^27^]), and variables for the global cognitive status (Mini‐Mental State Examination [MMSE]^28^; Parkinson Neuropsychometric Dementia Assessment [PANDA]^29^; cognitive status). All assessments were performed during ON medication state.

**TABLE 1:** Cognitive tests in the DEMPARK/LANDSCAPE study.

Theoretically assumed cognitive domain	Test score	Abbrevation
Attention	Digit Span forward^30^	DSfw
	Brief test of attention^31^	BTA
	Stroop word reading^32^	StrW
	Stroop color naming^32^	StrC
	Stroop interference^32^	StrI
Executive functions	Semantic Word Fluency^a^	SemWF
	Phonematic Word Fluency^a^	PhoWF
	Trail Making Test B/A^a^	TBA
	Digit Span backwards^30^	DSbw
	Modified Wisconsin Card Sorting Test categories^33^	CScat
	Modified Wisconsin Card Sorting Test non‐perservative errors^33^	CSnpe
	Modified Wisconsin Card Sorting Test perservative errors^33^	CSpe
Memory	Figures recall^a^	FigR
	Verbal Learning^a^	VbL
	Verbal Recall^a^	VbR
Language	Boston Naming Test^a^	BNT
Visuospatial functions	Figures copying^a^	FigC
	Leistungsprüfsystem 7^34^	LPS7
	Leistungsprüfsystem 9^34^	LPS9

^a^ Part of the extended Consortium to Establish a Registry for Alzheimer´s Disease (CERAD) neuropsychological test battery (CERAD+).^35^

### 2.3 Statistical analysis

Statistical analyses and data visualization were conducted in *R*.^36^ We provide the code used for the statistical analyses as Supporting Information. First, to test if the cognitive test data are suitable for estimating a network model, we applied the Loadings Comparison Test (LCT^37^), an algorithm based on neural networks that was trained to predict if data were generated from a latent factor model or a network model.

For dimensionality analysis, we applied EGA^11^ with the Louvain algorithm. EGA constitutes an expansion of the correlation matrix as a unidimensionality method^38^ to a glasso‐regularized gaussian graphical model (*tuning *= 0.5), using *R* package *EGAnet*.^39^ Further details including the code used for data analysis can be found as Supplementary Material. We then applied bootstrapping methods implemented within *EGAnet* (*bootEGA*, *N *= 5000 boots) to assess stability of the dimensionality analysis. To compare the EGA‐derived empirical dimensionality structure with the theoretically assumed domain structure in the DEMPARK/LANDSCAPE study,^24^ we computed the Total Entropy Fit Index (TEFI^40^) as well as model fit indices Chi^2^, Comparative Fit Index (CFI), and Root Mean Square Error of Approximation (RMSEA) based on CFA.

Following, as recommended to be used with EGA,^41^ we performed Unique Variable Analysis (UVA^42^) to identify possible redundancies in the test battery. UVA computes the *weighted topological overlap* (wTO), which “quantifies the extent to which a pair of nodes have (dis)similar connections”.^42^, ^43^ Variable pairs identified with wTO >0.25 indicated substantial redundancy^42^; thus we removed all but one variable from a set of redundant variables. Specifically, the variable showing the lowest maximum wTO with all other variables other than the one with which it is redundant is retained, whereas the other is removed.^42^ Finally, we re‐analyzed the now‐reduced set of cognitive test data applying EGA as described above.

### 2.4 Exploratory conceptual replication of findings in a sample of CHI

For an exploratory conceptual replication of our findings of EGA and UVA in the sample of individuals with PD from the DEMPARK/LANDSCAPE study, we used publicly available data of a large sample of CHIs compiled by Agelink van Rentergem et al.^17^ Following a systematic literature search, they performed a factor meta‐analysis with cognitive test data of *N *= 60,398 CHIs from 55 studies. All studies were published after 1997, and administered cognitive tests in a population of CHIs (i.e., without psychiatric or neurological disorders, disorders that could interfere with test administration, and conditions that were studied for their cognitive implications) without any further manipulations. The authors identified an overlap of 12 cognitive tests between the studies that were used for analysis (see Table S1). For this study, we used the total partial correlation matrix of the test variables that eliminated the influence of age, gender, and education from the data, as reported by Agelink van Rentergem et al.^21^ Based on this matrix, we applied EGA with the same configurations as in the analysis of individuals with PD from the DEMPARK/LANDSCAPE study. We then again performed UVA to identify and remove possible redundancy in the data and re‐performed EGA with reduced data.

## 3 RESULTS

### 3.1 Sample characteristics

In our sample of individuals with PD from the LANDSCAPE/DEMPARK baseline (*N* = 698), participants were *M *= 67.62 years old (SD = 7.99), 67.48% were male, and 32.52% were female. The sample included *n *= 282 individuals with PD‐NC, *n *= 314 individuals with PD‐MCI, and *n *= 102 individuals with PD‐D. For further information on the descriptive sample characteristics of sociodemographic, clinical, and cognitive variables, see Table 2.

**TABLE 2:** Descriptive sample characteristics.

	Individuals with PD *N* = 698
Age, years, mean (SD)	67.62 (7.88)
Gender, *n* (%)	
*Male*	471 (67.48%)
*Female*	227 (32.52%)
Years of education, mean (SD)	13.30 (3.18)
Disease duration in years, mean (SD)	6.78 (5.41)
Levodopa equivalent daily dose, mean (SD)	772.73 (553.31)
UPDRS‐III, mean (SD)	23.05 (12.28)
Hoehn & Yahr stages, *n* (%)	
*1*	102 (14.61%)
*2*	337 (48.28%)
*3*	198 (28.37%)
*4*	47 (6.73%)
*5*	10 (1.43%)
*Unknown*	4 (0.57%)
GDS, mean (SD)	3.48 (3.08)
MMSE, mean (SD)	27.89 (2.24)
PANDA, mean (SD)	21.45 (6.04)
Cognitive status, *n* (%)	
*PD‐NC*	282 (40.40%)
*PD‐MCI*	314 (44.99%)
*PD‐D*	102 (14.61%)

*Note*: Data are mean (SD) or *n* (%) as appropriate.

Abbreviations: GDS, Geriatric Depression Scale; MMSE, Mini‐Mental State Examination; PANDA, Parkinson Neuropsychometric Dementia Assessment; PD‐D, Parkinson's Disease dementia; PD‐MCI, Parkinson's Disease mild cognitive impairment; PD‐MCI, Parkinson's Disease normal cognition; UPDRS‐III, Unified Parkinson's Disease Rating Scale Part III.

### 3.2 EGA of cognitive test scores in PD

LCT predicted that the cognitive test data from the DEMPARK/LANDSCAPE study was generated from a network model rather than from a latent factor model, with 91.4% of bootstrapped replicate samples suggesting network models.

**FIGURE 1:**
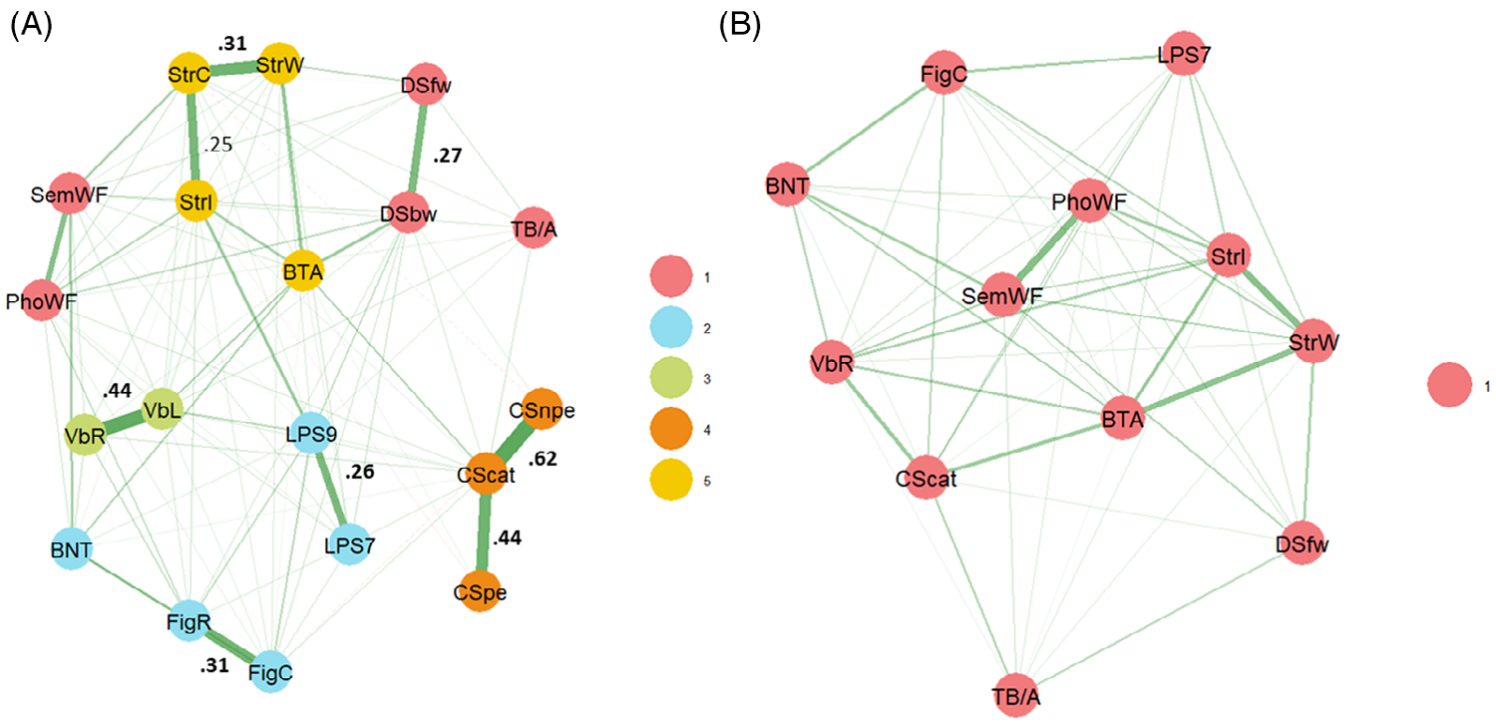
Network and dimensionality structure of cognitive functioning in individuals with PD (A) before and (B) after UVA. Green (solid) edges indicate positive pairwise conditional associations; red (dashed) edges indicate negative conditional associations. Node colors indicate assignment to dimensions as empirically derived by EGA. Values near edges indicate wTO, with wTO >0.20 indicating small‐to‐moderate redundancy, wTO >0.25 indicating moderate‐to‐large redundancy, and wTO >0.30 indicating large‐to‐very‐large redundancy. Values marked in bold indicate wTO is larger than the cutoff of wTO >0.25 for removal of redundant variables. BNT, Boston Naming Test; BTA, Brief Test of Attention; CScat, Modified Wisconsin Card Sorting Test categories; CSnpe, Modified Wisconsin Card Sorting Test non‐perservative errors; CSpe, Modified Wisconsin Card Sorting Test perservative errors; DSbw, Digit Span backwards; DSfw, Digit Span forward; EGA, exploratory graph analysis; FigC, Figures Copying; FigR, Figures Recall; LPS7, Leistungsprüfsystem 7; LPS9, Leistungsprüfsystem 9; PhoWF, phonematic Word Fluency; SemWF, semantic Word Fluency; StrC, Stroop color naming; StrI, Stroop interference; StrW, Stroop word reading; TB/A, Trail Making Test B/A; UVA, unique variable analysis; VbL, Verbal Learning; VbR, Verbal Recall; wTO, weighted topological overlap.

EGA identified five distinct dimensions that differed from the formerly theoretically assumed cognitive domain structure (Figure 1A). The result was confirmed by bootEGA in 64.92% of bootstrapped samples (see Figures S2 and S3 and Tables S2–S6 for network and dimensionality analysis stability results). Visual inspection of the estimated network model and the dimensionality analysis revealed that cognitive test scores originating from the same higher‐level test paradigm (e.g., Modified Wisconsin Card Sorting Test [MWCST]; Stroop test; Leistungsprüfsystem) were more strongly related to each other than to other tests/paradigms in the network. Dimensionality analysis did not separate scores from the same higher‐level test paradigm in any case, not even if those variables had been conceptually assumed to reflect different cognitive domains, namely Digit Span forward (attention) and Digit Span backwards (executive functions) or Figures Copying (visuospatial functions) and Figures Recall (memory).

Model fit indices of the empirical EGA‐derived structure and the theoretically assumed structure of cognitive domains are presented in Table 3.

**TABLE 3:** Model fit indices for empirical and theoretical structures.

	Model fit index			
Model	TEFI	Chi^2^	CFI	RMSEA
EGA	−9.54	526.98 *(satisfactory)*	0.92 *(satisfactory)*	0.06 *(poor)*
Theoretical	−7.86	896.72 *(satisfactory)*	0.78 *(poor)*	0.10 *(poor)*

*Note*: Interpretation in parentheses under values according to Byrne (1994).^44^ Lower TEFI values indicate better model fit. EGA = Structure derived from Exploratory Graph Analysis of cognitive test data of individuals with PD. Theoretical = Previously assumed structure of cognitive tests.^24^

Abbreviations: CFI, Comparative Fit Index; PD, Parkinson's disease; RMSEA, Root Mean Square Error of Approximation; TEFI, Total Entropy Fit Index.

### 3.3 UVA and re‐analysis

UVA revealed relevant topological overlap for several pairs of cognitive test scores (Figure 1A). To reduce redundancy within the pool of test scores, test scores for Verbal Learning, Figures Recall, MWCST perseverative errors and non‐perseverative errors, Stroop color naming, Leistungsprüfsystem 9, and Digit Span backwards were removed. EGA of reduced data suggested a unidimensional structure (Figure 1B).

### 3.4 Exploratory conceptual replication of findings in a sample of CHIs

**FIGURE 2:**
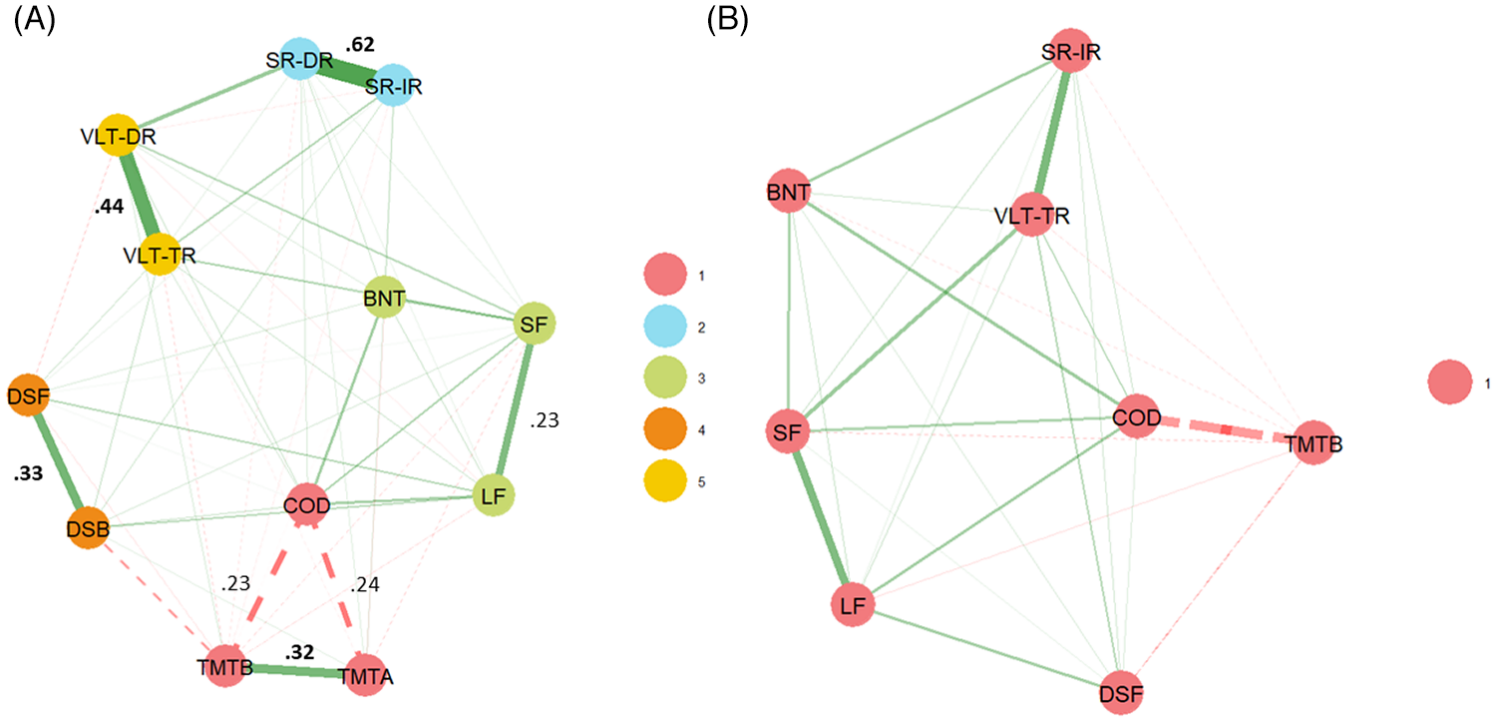
Network and dimensionality structure of cognitive functioning in CHIs (A) before and (B) after UVA. Green (solid) edges indicate positive pairwise conditional associations; red (dashed) edges indicate negative conditional associations. Node colors indicate assignment to dimensions as empirically derived by EGA. Values near edges indicate wTO, with wTO >0.20 indicating small‐to‐moderate redundancy, wTO >0.25 indicating moderate‐to‐large redundancy, and wTO >0.30 indicating large‐to‐very‐large redundancy. Values marked in bold indicate a wTO larger than the cutoff of wTO >0.25 for removal of redundant variables. BNT, Boston Naming Test; CHIs, cognitively healthy individuals; COD, Coding; DSB, Digit Span backwards; DSF, Digit Span forward; EGA, exploratory graph analysis; LF, Letter fluency; SF, Semantic Fluency; SR‐DR, Story Recall direct recall; SR‐IR, Story Recall immediate recall; TMTA, Trail Making Test part A; TMTB, Trail Making Test part B; UVA, unique variable analysis; VLT‐DR, Auditory Verbal Learning Test delayed recall; VLT‐TR, Auditory Verbal Learning Test total recall; wTO, weighted topological overlap.

EGA of cognitive test data in a group of CHIs identified five distinct dimensions (Figure 2A). The result was confirmed by bootEGA in 100% of bootstrapped samples (see Figure S4 and Table S7). Similar to the results from individuals with PD, test scores from the same higher‐level test paradigm were always categorized into the same dimensions. UVA revealed substantial redundancy within several pairs of cognitive test scores from the same higher‐level test paradigm (Figure 2A). After removal of the redundant variables Trail Making Test part A, Story Recall direct recall, Digit Span backwards, and Auditory Verbal Learning Test delayed recall, the EGA identified a unidimensional structure (Figure 2B).

## 4 DISCUSSION

In this study we analyzed the dimensionality structure of cognitive test scores in a large sample of individuals with PD from the DEMPARK/LANDSCAPE study with EGA, and compared the results to a theoretically assumed structure of cognitive domains. EGA identified five dimensions in a network model of cognitive test scores, which differed from the theoretically assumed cognitive domain structure. Specifically, EGA revealed close alignment of higher‐level test paradigms with empirically identified dimensions. Further analysis of the EGA‐derived structure revealed substantial overlap between test scores from the same higher‐level test paradigms, indicating a redundancy of single test scores within the cognitive test battery of the DEMPARK/LANDSCAPE study. Following data reduction, re‐performing EGA yielded a unidimensional structure. In an external validation based on a large CHI sample, we successfully conceptually replicated these findings.

Results showed that EGA‐derived dimensions were closely aligned with higher‐level test paradigms, indicating that these scores are even more closely associated with each other than test scores that were theoretically assigned to the same domain. Thus, for dimensionality analysis, originating from the same paradigm seems to be more influential than the theoretically assumed underlying cognitive domain. This finding aligns with the concept of method variance, which describes the tendency that variables from the same test correlate because their responses are elicited by the same stimuli or methodology.^8^, ^45^ It thus highlights the MDS guideline recommendation of not using two very similar test scores to evaluate cognitive performance within one cognitive domain.^5^ However, our results encourage expanding this recommendation even across theoretically assigned cognitive domains. As an example, test scores Figures Copying (FigC) and Figures Recall (FigR) are theoretically assumed to measure different cognitive domains (visuospatial functions and memory, respectively), but showed substantial overlap in UVA. As a practical implication, an individual presenting impairment in FigC due to impaired visuospatial functions would be likely to show impaired performance in FigR as well, mainly due to a large overlap of the general psychomotor abilities needed for successfully executing the task rather than additional memory deficits. Nevertheless, this individual would be diagnosed with multiple‐domain amnestic cognitive impairment according to standard diagnostic criteria. Hence, our results hint toward a possible inflation of multiple‐domain PD‐MCI diagnoses and, ultimately, limited validity of the PD‐MCI subtypes.

After removing redundant cognitive test scores identified by UVA, re‐analyzing the reduced data revealed a unidimensional structure, contradicting both the initially EGA‐derived empirical structure and the theoretically assumed structure of cognitive domains. This result might suggest that the concept of cognitive domains is reinforced by strong correlations between redundant test scores due to method variance, creating misleading microfactors.^42^ The unidimensional structure identified by UVA and EGA is in line with two common hypotheses on the structure of cognitive functions: the mutualism hypothesis of intelligence as well as the g‐factor hypothesis. The mutualism hypothesis explains positive intercorrelations between cognitive functions by reciprocal associations between cognitive process,^19^ not requiring a latent factor like the g‐factor.^18^ The LCT results of this study support the application of network perspectives of model cognitive functioning compared to a latent factor structure. It highlights network analysis and EGA as appropriate methods and suggests interpretation of our findings as evidence for the mutualism hypothesis rather than the g‐factor, although it remains compatible with both. Correspondingly, another study comparing psychometric network models with traditional FA cautiously interpreted their results in favor of the mutualism hypothesis.^46^

Even though this study is one of the first to use EGA to evaluate cognitive tests and, to the best of our knowledge, the first one to use it in a sample of individuals with PD, our findings are unlikely to be an artifact of the EGA method. That is because similar results have been observed in studies using traditional FA methods. First, a study that performed principal components FA to cognitive test data of individuals with PD‐MCI showed method variance as well, even if they were theoretically assumed to represent different cognitive domains: for example, Digit Span forward and Digit Span backwards.^47^ Second, in the factor meta‐analysis with cognitive test data of CHIs by van Rentergem et al.,^17^ the best fitting model consistently grouped test scores from the same test paradigm into the same factor too.

A limitation of this study is that our sample consisted of individuals with different cognitive statuses (PD‐NC, PD‐MCI, PD‐D). As it has been shown that different clinical groups show specific organizations of cognitive functions,^8^ this might be true for cognitive state subgroups within the same clinical group as well. Network neuropsychology studies have shown that cognitive organization can change with the onset of cognitive impairment, being reflected in network characteristics like node strength centrality.^14^, ^48^ Existing network neuropsychology studies aiming to identify communities within network models were heterogenous regarding statistical method, sample size, group of interest, and cognitive test scores included.^15^ Two studies applying EGA showed that the number of dimensions identified changed on the spectrum from groups of individuals with healthy cognition over SCD and MCI to AD.^13^, ^14^ However, results showed no consistent trend of an increasing or decreasing number of dimensions across this spectrum. These findings may be limited in generalizability and robustness, as they by far did not reach the recommended sample size of *n *= 500 to apply EGA for each group.^12^ To comply with this simulation‐based recommendation for sample size, we decided not to split the sample into subgroups of different cognitive statuses. Future studies should focus not only on cross‐sectional network and dimensionality analysis, but also on longitudinal approaches, for example, by applying dynamical EGA,^49^ which can perform longitudinal dimensionality analysis based on network models. This could help to gain a deeper understanding of how the organization of cognitive functioning may change over time.

Another limitation to consider is the exploratory and data‐driven approach applied in this study. Although it is possible to establish the stability of network analysis and EGA results by applying bootstrapping methods, it is not possible, to date, to establish the stability of UVA results. Hence, specific findings based on the UVA algorithm, for example, the degree of redundancy of specific variable pairs, need to be interpreted with caution. However, by conceptually replicating our findings in a different dataset with a different cognitive test battery, we provided evidence for the stability of the overarching finding that redundancy within cognitive test batteries could bias the empirical analysis of the cognitive functioning structure.

It is important to note that we also do not believe that our EGA results should be interpreted as the true structure of cognitive functions, as cognitive test scores do not necessarily reflect isolated cognitive functions. Thus, our findings might reflect a measurement problem regarding cognitive functions in general. Therefore, this issue is not limited to the network neuropsychology framework, but is also relevant to traditional FA methods. Furthermore, we would argue that criteria for cognitive diagnostics relying on cognitive test scores should consider the test score structure rather than a theoretical structure of cognitive functions. Hence, applying EGA, especially in combination with UVA, can be a useful tool for retrospectively evaluating the validity of cognitive test batteries. We believe that our retrospective analysis can influence future test battery compositions to improve valid and reliable measurement, reduce redundancy, and ensure the validity of cognitive diagnoses and their subtypes as we know them. Simultaneously, it also may pave the way for challenging the concept of cognitive domains and the diagnosis criteria that are associated with them in favor of concepts that reflect the complexity of cognition as an interconnected system. However, future research will need to develop appropriate test batteries, convenient parameters to evaluate cognitive performances, and adequate criteria for diagnosing cognitive impairment within this framework, which do not yet exist.

A strength of this study is that it is one of the first to systematically evaluate the structure of cognitive domains while taking a network perspective on cognition, especially in the groups of individuals with PD (and CHIs). Taking the first step to fill the gap in this under‐investigated field, we applied an exploratory dimensionality analysis approach and integrated confirmatory approaches by comparing two empirically derived structure with a prior theoretically assumed structure using CFA. Future studies aiming to replicate our findings should ideally be preregistered with clear hypotheses to further improve confidence in these findings.

We believe that one major strength of this study is combining network and dimensionality analysis by applying EGA. As we already mentioned, theoretical^19^ as well as empirical aspects (results of the LCT) support the view of cognition as a complex network of interacting cognitive functions. To make inferences about complex interconnected systems like cognition, we need to apply methods that can adequately reflect this complexity, like network analysis methods.^16^ Another strength is that we were able to conceptually replicate our findings in another, independent, large sample of CHIs,^21^ providing first evidence that our results are conceptually replicable and generalizable, and not specific to PD. Furthermore, analyses were based on demographically adjusted, standardized *z*‐scores in the PD sample and by partial correlation in the CHI sample. Even though demographical adjustments differed between cognitive test scores, there were no hints that results were biased by the influences of demographical variables.

## 5 CONCLUSION

To conclude, by combining network and dimensionality analysis by applying EGA to cognitive test scores of almost 700 individuals with PD, we found that theoretically assumed categorizations of cognitive test scores into cognitive domains could not be replicated. Furthermore, results showed a high redundancy between test scores, having profound implications for the validity of PD‐MCI diagnoses and subtype classifications, as well as the composition of cognitive test batteries in this context. Conceptually replicating the results in a large sample of CHIs, we interpret our findings in the light of the emerging field of network neuropsychology, arguing that cognitive functioning can be viewed as a complex network of interconnected cognitive functions rather than a latent factor structure with modular cognitive domains.

## CONFLICT OF INTEREST STATEMENT

D.S., M.B.G., D.B., R.H.R., B.M., K.R., O.R., S.R., J.B.S., A.S., C.T., H.U.W., R.D., and A.O. have no competing interests to declare. E.K. received grants from the German Ministry of Education and Research, the Joint Federal Committee, and The German Parkinson Foundation, all outside the submitted work. E.K. received honoraria from the companies EISAI GmbH, Germany, memodio GmbH, Germany, Desitin GmbH, Germany, and Prolog GmbH, Germany, all outside the submitted work. J.K. is a consultant and speaker for AbbVie, Bial, Biogen, Desitin, Esteve, Novartis, Roche, STADA, UCB Pharma, and Zambon; in addition, he is Specialty Chief Editor for *Frontiers in Neurology* (section Applied Neuroimaging) and Associate Editor (Neurology) for *Therapeutic Advances in Chronic Disease*. I.L.S. reports funding from Bayer AG and travel grants from Desitin and the German Society for Neurology outside the submitted work. K.W. receives funding from the Deutsche Forschungsgemeinschaft (German Research Association) and STADAPHARM GmbH outside the present study. He has received honoraria for presentations/advisory boards/consultations from BIAL, Indorsia, Boston Scientific and STADAPHARM GmbH, outside the present study. He has received royalties from Thieme Press and Elsevier Press. He serves as an editorial board member of Wiley's *Parkinson's Disease*, *Behavioural Neurology*, and *PLOS One*. Author disclosures are available in the Supporting Information.

## CONSENT STATEMENT

The authors assert that all procedures contributing to this work comply with the ethical standards of the relevant national and institutional committees on human experimentation and with the Helsinki Declaration of 1975, as revised in 2008. All study procedures of the DEMPARK/LANDSCAPE project were approved by the Ethics Committee of Philipps University Marburg (approval‐no. 178/07) in March 2009, and thereupon by the ethics committees of the participating centers. All participants gave written informed consent prior to their inclusion in the study.

## Supporting information



Supporting Information

Supporting Information

Supporting Information

## References

[1] Aarsland D , Batzu L , Halliday GM , et al. Parkinson disease‐associated cognitive impairment. Nature Reviews Disease Primers. 2021;7(1):47. 10.1038/s41572-021-00280-3 34210995

[2] Bloem BR , Okun MS , Klein C . Parkinson's disease. Lancet. 2021;397(10291):2284‐303. 33848468 10.1016/S0140-6736(21)00218-X

[3] Baiano C , Barone P , Trojano L , Santangelo G . Prevalence and clinical aspects of mild cognitive impairment in Parkinson's disease: a meta‐analysis. Mov Disord. 2020;35(1):45‐54. 31743500 10.1002/mds.27902

[4] Emre M , Aarsland D , Brown R , et al. Clinical diagnostic criteria for dementia associated with Parkinson's disease. Mov Disord. 2007;22(12):1689‐1707. 17542011 10.1002/mds.21507

[5] Litvan I , Goldman JG , Tröster AI , et al. Diagnostic criteria for mild cognitive impairment in Parkinson's disease: movement disorder society task force guidelines. Mov Disord. 2012;27(3):349‐356. 22275317 10.1002/mds.24893 PMC3641655

[6] Wood K‐L , Myall DJ , Livingston L , et al. Different PD‐MCI criteria and risk of dementia in Parkinson's disease: 4‐year longitudinal study. NPJ Parkinson's disease. 2016;2:15027. 10.1038/npjparkd.2015.27 PMC5516585 28725690

[7] American Psychiatric Association . Diagnostic and Statistical Manual of Mental Disorders. 5th ed. American Psychiatric Association; 2013.

[8] Delis DC , Jacobson M , Bondi MW , Hamilton JM , Salmon DP . The myth of testing construct validity using factor analysis or correlations with normal or mixed clinical populations: lessons from memory assessment. J Int Neuropsychol Soc. 2003;9(6):936‐946. 14632252 10.1017/S1355617703960139

[9] Chung SJ , Lee HS , Kim H‐R , et al. Factor analysis‐derived cognitive profile predicting early dementia conversion in PD. Neurology. 2020;95(12):e1650‐e1659. 32651296 10.1212/WNL.0000000000010347

[10] Yang C , Garrett‐Mayer E , Schneider JS , Gollomp SM , Tilley BC . Repeatable battery for assessment of neuropsychological status in early Parkinson's disease. Mov Disord. 2009;24(10):1453‐1460. 19452561 10.1002/mds.22552 PMC3915413

[11] Golino H , Epskamp S . Exploratory graph analysis: a new approach for estimating the number of dimensions in psychological research. PloS One. 2017;12(6):e0174035. 28594839 10.1371/journal.pone.0174035 PMC5465941

[12] Golino H , Shi D , Christensen AP , et al. Investigating the performance of exploratory graph analysis and traditional techniques to identify the number of latent factors: a simulation and tutorial. Psychol Methods. 2020;25(3):292‐320. 32191105 10.1037/met0000255 PMC7244378

[13] Tosi G , Nigro S , Urso D , et al. The network structure of cognitive impairment: from subjective cognitive decline to Alzheimer's disease. J Neurosc. 2025;45(2):e2082242024. 10.1523/JNEUROSCI.2082-24.2024 PMC11714334 39715691

[14] Grunden N , Phillips NA . A network approach to subjective cognitive decline: exploring multivariate relationships in neuropsychological test performance across Alzheimer's disease risk states. Cortex. 2024;173:313‐332. 38458017 10.1016/j.cortex.2024.02.005

[15] Ferguson CE . Network neuropsychology: the map and the territory. Neurosc Biobehav Rev. 2022;132:638‐647. 10.1016/j.neubiorev.2021.11.024 34800585

[16] Borsboom D , Deserno MK , Rhemtulla M , et al. Network analysis of multivariate data in psychological science. Nat Rev Methods Primers. 2021;1(1):1.

[17] Wang Z , Xin J , Wang Z , Yao Y , Zhao Y , Qian W . Brain functional network modeling and analysis based on fMRI: a systematic review. Cog Neurodyn. 2021;15(3):389‐403. 10.1007/s11571-020-09630-5 PMC8131458 34040667

[18] Spearman C . The abilities of man. Macmillan; 1927.

[19] van der Maas HLJ , Dolan CV , Grasman RPPP , Wicherts JM , Huizenga HM , Raijmakers MEJ . A dynamical model of general intelligence: the positive manifold of intelligence by mutualism. Psychol Rev. 2006;113(4):842‐61. 17014305 10.1037/0033-295X.113.4.842

[20] Balzer‐Geldsetzer M , Da Costa ASFB , Kronenbürger M , et al. Parkinson's disease and dementia: a longitudinal study (DEMPARK). Neuroepidemiology. 2011;37(3‐4):168‐176. 22067139 10.1159/000331490

[21] Agelink van Rentergem JA , Vent NR de , Schmand BA , Murre JMJ , Staaks JPC , Huizenga HM . The factor structure of cognitive functioning in cognitively healthy participants: a meta‐analysis and meta‐analysis of individual participant data. Neuropsychol Rev. 2020;30(1):51‐96. 32008158 10.1007/s11065-019-09423-6 PMC7089912

[22] Emre M . Dementia associated with Parkinson's disease. Lancet Neurol. 2003;2(4):229‐237. 12849211 10.1016/s1474-4422(03)00351-x

[23] Petersen RC . Mild cognitive impairment as a diagnostic entity. J Intern Med. 2004;256(3):183‐194. 15324362 10.1111/j.1365-2796.2004.01388.x

[24] Kalbe E , Rehberg SP , Heber I , et al. Subtypes of mild cognitive impairment in patients with Parkinson's disease: evidence from the LANDSCAPE study. J Neurol Neurosurg Psychiatry. 2016;87(10):1099‐105. 27401782 10.1136/jnnp-2016-313838

[25] Fahn SM . Unified Parkinson's disease rating scale: In: Fahn SM , Goldstein M , Calne DB , editors. Recent developments in Parkinson's Disease. Florham Park: Macmillan Healthcare Information; 1987:153‐63.

[26] Hoehn MM , Yahr MD . Parkinsonism: onset, progression and mortality. Neurology. 1967;17(5):427‐42. 6067254 10.1212/wnl.17.5.427

[27] Yesavage JA , Brink TL , Rose TL , et al. Development and validation of a geriatric depression screening scale: a preliminary report. J Psychiatr Res. 1982;17(1):37‐49. 7183759 10.1016/0022-3956(82)90033-4

[28] Folstein MF , Folstein SE , McHugh PR . “Mini‐mental state”. A practical method for grading the cognitive state of patients for the clinician. J Psychiatr Res. 1975;12(3):189‐98. 1202204 10.1016/0022-3956(75)90026-6

[29] Kalbe E , Calabrese P , Kohn N , et al. Screening for cognitive deficits in Parkinson's disease with the Parkinson neuropsychometric dementia assessment (PANDA) instrument. Parkinsonism Relat Disord. 2008;14(2):93‐101. 17707678 10.1016/j.parkreldis.2007.06.008

[30] Härting C , Neufeld H , Calabrese P , Deisinger K , Kessler J . Wechsler Memory Scale revised version. WMS‐R; 2000.

[31] Schretlen D , Bobholz JH , Brandt J . Development and psychometric properties of the brief test of attention. The Clinical Neuropsychologist 1996;10(1):80‐9.

[32] Bäumler G . Farbe‐Wort‐Interferenztest (FWIT) nach JR Stroop. Hogrefe Verlag für Psychologie; 1985.

[33] Nelson HE . A modified card sorting test sensitive to frontal lobe defects. Cortex. 1976;12(4):313‐324. 1009768 10.1016/s0010-9452(76)80035-4

[34] Sturm W , Horn W , Willmes K . Leistungsprüfsystem für 50‐90‐Jährige (LPS 50+). Hogrefe Verlag für Psychologie; 1993.

[35] Schmid NS , Ehrensperger MM , Berres M , Beck IR , Monsch AU . The extension of the German CERAD neuropsychological assessment battery with tests assessing subcortical, executive and frontal functions improves accuracy in dementia diagnosis. Dementia and Geriatric Cognitive Disorders Extra. 2014;4(2):322‐334. 25298776 10.1159/000357774 PMC4176468

[36] R Core Team . R: a language and environment for statistical computing. Vienna, Austria: R Foundation for Statistical Computing; 2024.

[37] Christensen AP , Golino H . Factor or network model? Predictions from neural networks. JBDS. 2021;1(1):85‐126.

[38] Christensen AP , Garrido LE , Guerra‐Peña K , Golino H . Comparing community detection algorithms in psychometric networks: A Monte Carlo simulation. Behav Res Meth. 2024;56(3):1485‐1505. 10.3758/s13428-023-02106-4 37326769

[39] Golino H , Christensen AP . EGAnet: Exploratory Graph Analysis ‐ A framework for estimating the number of dimensions in multivariate data using network psychometrics; 2024.

[40] Golino H , Moulder R , Shi D , et al. Entropy fit indices: new fit measures for assessing the structure and dimensionality of multiple latent variables. Multivariate Behav Res. 2021;56(6):874‐902. 32634057 10.1080/00273171.2020.1779642

[41] Golino H , Christensen AP . Exploratory Graph Analysis. [November 22, 2024]. Available from: https://r‐ega.net/articles/ega.html

[42] Christensen AP , Garrido LE , Golino H . Unique variable analysis: a network psychometrics method to detect local dependence. Multivariate Behav Res. 2023;58(6):1165‐1182. 37139938 10.1080/00273171.2023.2194606

[43] Ravasz E , Somera AL , Mongru DA , Oltvai ZN , Barabási AL . Hierarchical organization of modularity in metabolic networks. Science. 2002;297(5586):1551‐5. 12202830 10.1126/science.1073374

[44] Byrne BM . Structural Equation Modeling with EQS and EQS/WINDOWS: Basic Concepts, Applications, and Programming: SAGE; 1994.

[45] Larrabee GJ . Lessons on measuring construct validity: a commentary on Delis, Jacobson, Bondi, Hamilton, And Salmon. Journal of the International Neuropsychological Society JINS. 2003;9(6):947‐953. 14632253 10.1017/S1355617703960140

[46] Kan K‐J , van der Maas HL , Levine SZ . Extending psychometric network analysis: empirical evidence against g in favor of mutualism?. Intelligence. 2019;73:52‐62.

[47] Cholerton BA , Zabetian CP , Wan JY , et al. Evaluation of mild cognitive impairment subtypes in Parkinson's disease. Mov Disord. 2014;29(6):756‐64. 24710804 10.1002/mds.25875 PMC4013249

[48] Ferguson CE . A network psychometric approach to neurocognition in early Alzheimer's disease. Cortex. 2021;137:61‐73. 33607345 10.1016/j.cortex.2021.01.002

[49] Golino H , Christensen AP , Moulder R , Kim S , Boker SM . Modeling latent topics in social media using dynamic exploratory graph analysis: the case of the right‐wing and left‐wing trolls in the 2016 US elections. Psychometrika. 2022;87(1):156‐187. 34757581 10.1007/s11336-021-09820-y PMC9021116

