# Supplementary material for: A network perspective on cognition in individuals with Parkinson's disease
Source: Alzheimers Dement (Amst). 2025 Feb 24;17(1):e70091. doi: 10.1002/dad2.70091 (PMC11848640; doi:10.1002/dad2.70091)
Supplement: Supplementary file 1 — Supporting Information [file DAD2-17-e70091-s003.docx]

**SUPPLEMENTARY MATERIAL**

**This file contains Supplementary Material to**

Scharfenberg, D., Kalbe, E., Balzer-Geldsetzer, M., Berg, D., Hilker-Roggendorf, R., Kassubek, J., Liepelt-Scarfone, I., Mollenhauer, B., Reetz, K., Riedel, O., Roeske, S., Schulz, J. B., Storch, A., Trenkwalder, C., Witt, K., Wittchen, H.‑U., Dodel, R., & Ophey, A. (2025). A Network Perspective on Cognition in Individuals with Parkinson’s Disease. Alzheimer’s & Dementia: Diagnosis, Assessment & Disease Monitoring. <https://doi.org/10.1002/dad2.70091>

**Corresponding authors**:

Daniel Scharfenberg

Medical Psychology | Neuropsychology and Gender Studies, Center for Neuropsychological Diagnostics and Intervention (CeNDI), University Hospital Cologne and Faculty of Medicine, University of Cologne, Cologne, Germany

Kerpener Str. 62, 50937 Köln

Phone: +49 221 478-86485

[daniel.scharfenberg@uk-koeln.de](mailto:daniel.scharfenberg@uk-koeln.de)

Prof. Elke Kalbe
Medical Psychology | Neuropsychology and Gender Studies, Center for Neuropsychological Diagnostics and Intervention (CeNDI), University Hospital Cologne and Faculty of Medicine, University of Cologne, Cologne, Germany
Kerpener Str. 62, 50937 Köln
Phone: +49 221 478-96244
elke.kalbe@uk-koeln.de

**SM Figure 1** *Descriptive statistics and distribution of cognitive test data in the LANDSCAPE study.*

**
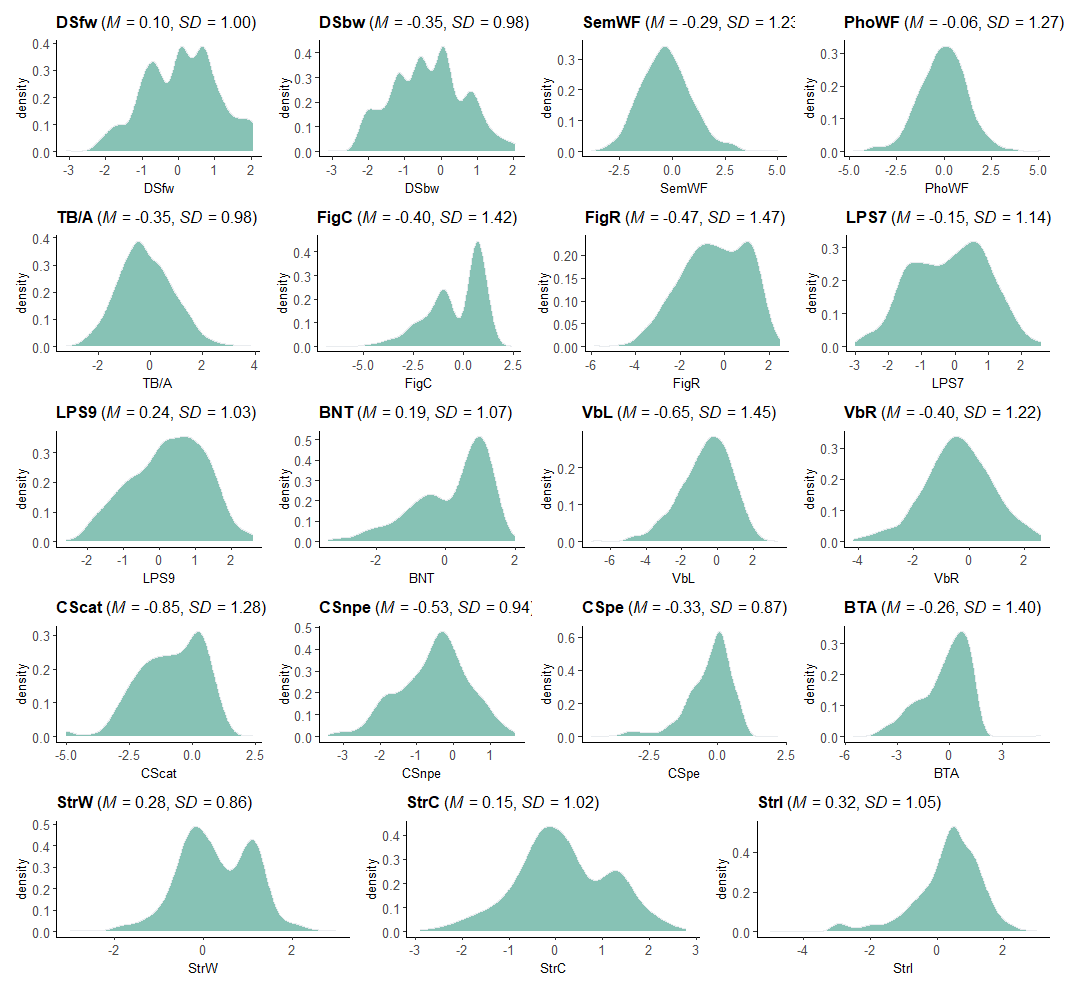
**

*Note*. DSfw = Digit Span forward; DSbw = Digit Span backwards; SemWF = semantic word fluency; PhoWF = phonematic word fluency; TB/A = Trail Making Test B/A; FigC = Figures Copying; FigR = Figures Recall; LPS7 = Leistungsprüfsystem 7; LPS9 = Leistungsprüfsystem 9; BNT = Boston Naming Test; VbL = verbal learning; VbR = verbal recall; CScat = Modified Wisconsin Card Sorting Test categories; CSnpe = Modified Wisconsin Card Sorting Test non-perservative errors; CSpe = Modified Wisconsin Card Sorting Test perservative errors; BTA = Brief Test of Attention; StrW = Stroop word reading; StrC = Stroop color naming; StrI = Stroop interference.

**SM Table 1***. Cognitive tests used for data analysis in cognitively healthy individuals.*

| *Test variable* | *Abbreviation* | *Additional information* |
| --- | --- | --- |
| Trail Making Test Part A | TMTA | Combined with Color Trails Test Part 1, D-KEFS Trail Making Test condition 2. |
| Trail Making Test Part B | TMTB | Combined with Color Trails Test Part 2, D-KEFS Trail Making Test condition 4. |
| Story Recall Immediate Recall | SR-IR | Combined across multiple WMS Logical Memory versions, combined with RBANS Story Immediate Memory. |
| Story Recall Delayed Recall | SR-DR | Combined across multiple WMS Logical Memory versions, combined with RBANS Story Delayed Memory. |
| Letter Fluency | LF | Synonyms: Controlled Oral Word Association Test, Phonemic Verbal Fluency. |
| Semantic Fluency | SF | Synonyms: Categorical Verbal Fluency. Preferential inclusion of the “Animals” version if multiple were available. |
| Digit Span Forward | DSF | Combined across multiple WAIS and WMS versions. |
| Digit Span Backwards | DSB | Combined across multiple WAIS and WMS versions. |
| Coding | COD | Combined across multiple WAIS versions. Synonym: Digit Symbol Substitution. |
| Boston Naming Test | BNT |  |
| Auditory Verbal Learning Test – Total Recall | VLT-TR | Combined with California Verbal Learning Test – Total Recall, the Hopkins Verbal Learning Test – Total Recall, and RBANS List Learning. |
| Auditory Verbal Learning Test – Delayed Recall | VLT-DR | Combined with California Verbal Learning Test – Long-Delay Recall, the Hopkins Verbal Learning Test – Delayed Recall, and RBANS List Recall. |

*Note.* “Table 1: Included test variables” from van Rentergem et al. (2020) published under Creative Commons Attribution 4.0 International License (http://creativecommons.org/licenses/by/4.0/).

**SM Table 2.** *CS-coefficients of network structure of cognitive functioning in individuals with Parkinson’s disease.*

|  | Edge-weight | Node strength |
| --- | --- | --- |
| CS-coefficient | .751 | .751 |

*Note*. CS-coefficient = correlation stability coefficient, indicating the per the percentage of cases in the total sample that can be dropped while retaining a correlation of about 0.7 between the order of the centrality index in the subsampled data network and the order of the centrality index in the full data network. CS-coefficient should not be below 0.25 and preferably above 0.5.

**SM Figure 2.** *Network and dimensionality structure of cognitive functioning in individuals with Parkinson’s Disease as derived by bootEGA.*

*
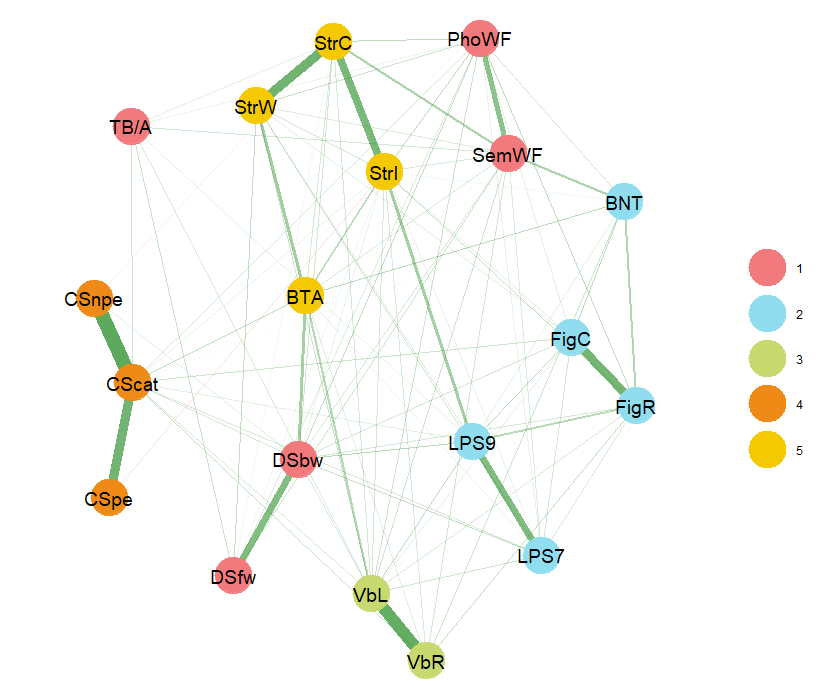
*

*Note*. *N* = 5000 boots. Green edges indicate positive pairwise conditional associations; red edges indicate negative conditional associations. Node colors indicate assignment to dimensions as empirically derived by Exploratory Graph Analysis.
 DSfw = Digit Span forward; DSbw = Digit Span backwards; StrW = Stroop word reading; StrC = Stroop color naming; StrI = Stroop interference; BTA = Brief Test of Attention; PhoWF = phonematic word fluency; SemWF = semantic word fluency; BNT = Boston Naming Test; FigC = Figures Copying; FigR = Figures Recall; LPS7 = Leistungsprüfsystem 7; LPS9 = Leistungsprüfsystem 9; VbL = verbal learning; VbR = verbal recall; TB/A = Trail Making Test B/A; CSnpe = Modified Wisconsin Card Sorting Test non-perservative errors; CScat = Modified Wisconsin Card Sorting Test categories; CSpe = Modified Wisconsin Card Sorting Test perservative errors.

**SM Table 3.** *Relevant network loadings of the bootEGA dimensionality analysis.*

|  | Dimension | | | | |
| --- | --- | --- | --- | --- | --- |
| Test score | 1 | 2 | 3 | 4 | 5 |
| DSfw | **.255** |  |  |  |  |
| DSbw | **.237** | *.116* |  |  | *.102* |
| SemWF | **.213** |  |  |  | *.103* |
| PhoWF | **.203** |  |  |  | .104 |
| TB/A | **.060*** |  |  |  |  |
| FigC |  | **.321** |  |  |  |
| FigR |  | **.330** |  |  |  |
| LPS7 |  | **.205** |  |  |  |
| LPS9 |  | **.271** |  |  |  |
| BNT |  | **.110** |  |  |  |
| VbL |  |  | **.41** |  | *.102* |
| VbR |  |  | **.41** |  |  |
| CScat |  |  |  | **.678** |  |
| CSnpe |  |  |  | **.399** |  |
| CSpe |  |  |  | **.279** |  |
| BTA |  |  |  |  | **.158** |
| StrW | *.141* |  |  |  | **.333** |
| StrC |  |  |  |  | **.437** |
| StrI |  | *.121* |  |  | **.268** |

*Note.* The table shows relevant network loadings (< .100). Values marked in bold indicate that the corresponding test score was assigned to the corresponding dimension by bootstrapped Exploratory Graph Analysis (*N* = 5000 boots). Values marked in italics indicate potential cross-loadings with other dimensions.

DSfw = Digit Span forward; DSbw = Digit Span backwards; SemWF = semantic word fluency; PhoWF = phonematic word fluency; TB/A = Trail Making Test B/A; FigC = Figures Copying; FigR = Figures Recall; LPS7 = Leistungsprüfsystem 7; LPS9 = Leistungsprüfsystem 9; BNT = Boston Naming Test; VbL = verbal learning; VbR = verbal recall; CScat = Modified Wisconsin Card Sorting Test categories; CSnpe = Modified Wisconsin Card Sorting Test non-perservative errors; CSpe = Modified Wisconsin Card Sorting Test perservative errors; BTA = Brief Test of Attention; StrW = Stroop word reading; StrC = Stroop color naming; StrI = Stroop interference.

* TB/A was assigned to dimension 1, although showing no network loading > .100.

**SM Table 4***.* *Frequency of identified number of dimensions in bootEGA.*

| Number of Dimensions | 4 | 5 | 6 | 7 |
| --- | --- | --- | --- | --- |
| Frequency | 0.0138 | 0.6492 | 0.3324 | 0.0046 |

*Note*. N = 5000 boots.

**SM Table 5***. Structural consistency of the dimensions identified in EGA.*

| Dimension | 1 | 2 | 3 | 4 | 5 |
| --- | --- | --- | --- | --- | --- |
| Frequency | .2742 | .3276 | .9756 | 1.000 | .9120 |

*Note*. Frequency = The proportion of times that each empirical EGA dimension exactly replicated across the bootEGA samples (*N* = 5000 boots).

**SM Figure 3***. Item stability of cognitive test scores included in EGA.*


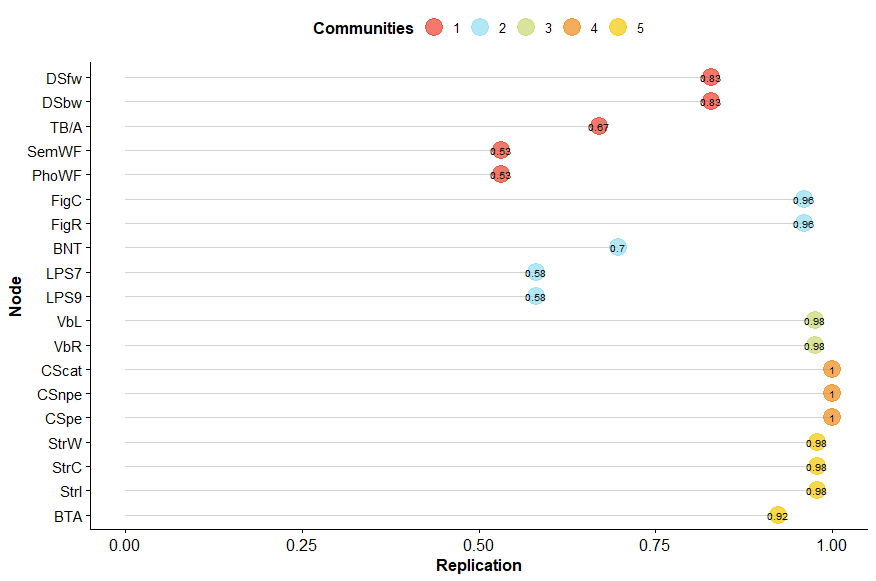


*Note*. Frequencies indicate the number of times a variable is estimated in the same dimension as by the empirical EGA structure based on bootEGA (*N* = 5000 boots). DSfw = Digit Span forward; DSbw = Digit Span backwards; StrW = Stroop word reading; StrC = Stroop color naming; StrI = Stroop interference; BTA = Brief Test of Attention; PhoWF = phonematic word fluency; SemWF = semantic word fluency; BNT = Boston Naming Test; FigC = Figures Copying; FigR = Figures Recall; LPS7 = Leistungsprüfsystem 7; LPS9 = Leistungsprüfsystem 9; VbL = verbal learning; VbR = verbal recall; TB/A = Trail Making Test B/A; CSnpe = Modified Wisconsin Card Sorting Test non-perservative errors; CScat = Modified Wisconsin Card Sorting Test categories; CSpe = Modified Wisconsin Card Sorting Test perservative errors.

**SM Table 6***. Item structural stability of cognitive test scores included in EGA.*

|  | Dimension | | | | | | |
| --- | --- | --- | --- | --- | --- | --- | --- |
| Test score | 1 | 2 | 3 | 4 | 5 | 6 | 7 |
| SemWF | 0.5318 | 0.1844 | 0.0000 | 0.0000 | 0.2178 | 0.0660 | 0.0000 |
| BNT | 0.1946 | 0.6978 | 0.0026 | 0.0000 | 0.0518 | 0.0532 | 0.0000 |
| VbL | 0.0070 | 0.0036 | 0.9756 | 0.0000 | 0.0002 | 0.0136 | 0.0000 |
| VbR | 0.0070 | 0.0036 | 0.9756 | 0.0000 | 0.0002 | 0.0136 | 0.0000 |
| FigC | 0.0374 | 0.9598 | 0.0000 | 0.0000 | 0.0000 | 0.0028 | 0.0000 |
| FigR | 0.0374 | 0.9598 | 0.0000 | 0.0000 | 0.0000 | 0.0028 | 0.0000 |
| PhoWF | 0.5318 | 0.1842 | 0.0000 | 0.0000 | 0.2180 | 0.0660 | 0.0000 |
| TB/A | 0.6704 | 0.0418 | 0.0790 | 0.1056 | 0.0758 | 0.0274 | 0.0000 |
| CScat | 0.0000 | 0.0000 | 0.0000 | 1.0000 | 0.0000 | 0.0000 | 0.0000 |
| CSnpe | 0.0000 | 0.0000 | 0.0000 | 1.0000 | 0.0000 | 0.0000 | 0.0000 |
| CSpe | 0.0000 | 0.0000 | 0.0000 | 1.0000 | 0.0000 | 0.0000 | 0.0000 |
| BTA | 0.0620 | 0.0004 | 0.0054 | 0.0000 | 0.9230 | 0.0092 | 0.0000 |
| StrW | 0.0220 | 0.0000 | 0.0000 | 0.0000 | 0.9780 | 0.0000 | 0.0000 |
| StrC | 0.0220 | 0.0000 | 0.0000 | 0.0000 | 0.9780 | 0.0000 | 0.0000 |
| StrI | 0.0220 | 0.0000 | 0.0000 | 0.0000 | 0.9780 | 0.0000 | 0.0000 |
| LPS7 | 0.1786 | 0.5808 | 0.0000 | 0.0000 | 0.0056 | 0.2336 | 0.0014 |
| LPS9 | 0.1786 | 0.5808 | 0.0000 | 0.0000 | 0.0056 | 0.2336 | 0.0014 |
| DSfw | 0.8292 | 0.0092 | 0.0000 | 0.0000 | 0.0300 | 0.1278 | 0.0038 |
| DSbw | 0.8292 | 0.0092 | 0.0000 | 0.0000 | 0.0300 | 0.1278 | 0.0038 |

*Note*. Frequencies indicate variables’ replication frequency, i.e., proportion of bootstraps that a variable appeared in each dimension based on bootEGA (*N* = 5000 boots). DSfw = Digit Span forward; DSbw = Digit Span backwards; StrW = Stroop word reading; StrC = Stroop color naming; StrI = Stroop interference; BTA = Brief Test of Attention; PhoWF = phonematic word fluency; SemWF = semantic word fluency; BNT = Boston Naming Test; FigC = Figures Copying; FigR = Figures Recall; LPS7 = Leistungsprüfsystem 7; LPS9 = Leistungsprüfsystem 9; VbL = verbal learning; VbR = verbal recall; TB/A = Trail Making Test B/A; CSnpe = Modified Wisconsin Card Sorting Test non-perservative errors; CScat = Modified Wisconsin Card Sorting Test categories; CSpe = Modified Wisconsin Card Sorting Test perservative errors.

**SM Figure 4***. Network and dimensionality structure of cognitive functioning in cognitively healthy individuals as derived by bootEGA.*


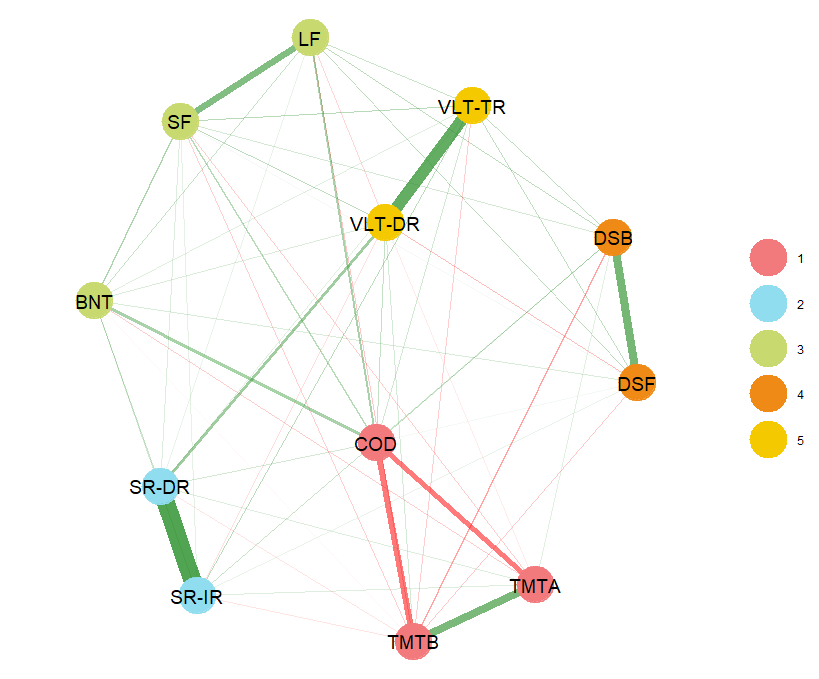


*Note. N* = 5000 boots. Green edges indicate positive pairwise conditional associations; red edges indicate negative conditional associations. Node colors indicate assignment to dimensions as empirically derived by Exploratory Graph Analysis.
SR-DR = Story Recall Direct Recall; SR-IR = Story Recall Immediate Recall; BNT = Boston Naming Test; SF = Semantic Fluency; LF = Letter Fluency; COD = Coding; TMTA = Trail Making Test Part A; TMTB = Trail Making Test Part B; DSF = Digit Span forward; DSB = Digit Span backwards; VLT-DR = Auditory Verbal Learning Test Delayed Recall; VLT-TR = Auditory Verbal Learning Test Total Recall.

**SM Table 7**. *Relevant network loadings of the bootEGA dimensionality analysis.*

|  | Dimension | | | | |
| --- | --- | --- | --- | --- | --- |
| Test score | 1 | 2 | 3 | 4 | 5 |
| TMTA | **.388** |  |  |  |  |
| TMTB | **.391** |  | *-.121* |  |  |
| COD | **-.319** |  | *.245* |  |  |
| SR-IR |  | **.548** |  |  |  |
| SR-DR |  | **.548** |  |  | *.128* |
| SF |  |  | **.304** |  | .114 |
| LF |  |  | **.262** | *.133* |  |
| BNT | *-.100* |  | **.119** |  |  |
| DSF |  |  |  | **.332** |  |
| DSB | *-.107* |  |  | **.332** |  |
| VLT-TR |  |  | *.105* | *.112* | **.399** |
| VLT-DR |  | *.124* |  |  | **.399** |

*Note.* The table shows relevant network loadings (< .100). Values marked in bold indicate that the corresponding test score was assigned to the corresponding dimension by bootEGA. Values marked in italics indicate potential cross-loadings with other dimensions.
SR-DR = Story Recall Direct Recall; SR-IR = Story Recall Immediate Recall; BNT = Boston Naming Test; SF = Semantic Fluency; LF = Letter Fluency; COD = Coding; TMTA = Trail Making Test Part A; TMTB = Trail Making Test Part B; DSF = Digit Span forward; DSB = Digit Span backwards; VLT-DR = Auditory Verbal Learning Test Delayed Recall; VLT-TR = Auditory Verbal Learning Test Total Recall.
